# Supplementary material for: AI-IoT Low-Cost Pollution-Monitoring Sensor Network to Assist Citizens with Respiratory Problems
Source: Sensors (Basel). 2023 Dec 3;23(23):9585. doi: 10.3390/s23239585 (PMC10708678; doi:10.3390/s23239585)
Supplement: Supplementary file 1 [file sensors-23-09585-s001.zip › sensors-2711839-supplementary.pdf]

Supplementary Materials:

Table S1. List of the most harmful air pollutants wit a brief description and their maximum values recommended according to WHO Global Air Quality Guidelines. 2023. Available online: https://apps.who.int/iris/bitstream/handle/10665/345329/9789240034228-eng.pdf (accessed on 15 March 202).

PM	are particles of microscopic size, from different components such as sulphate, nitrates, sodium chloride, mineral dust, to name a few. These particles can have different sizes, with different diameters, usually 1.0, 2.5 and 10 um. These particles can penetrate deep into the lungs and bloodstream, causing cardiovascular and respiratory diseases. Long-term exposure has been related to adverse perinatal outcomes and lung cancer. The maximum in average recommended is 15 and 45 ug/m3 in a day for PM2.5 and PM10 respectively.
CO	is a toxic gas without color, odor and taste. It makes difficult the body's cells to get oxygen, causing difficulties breathing. The maximum in average recommended is 4 ug/m3 in a day.
O3	or ground level ozone, is formed through the reaction with gases (such as VOCs, CO, and NO2) in the presence of sunlight at ground level. It can cause problems breathing, asthma and lung diseases. The maximum in average recommended is 100 ug/m3 in 8 hours.
NO2	is a gas commonly produced in the combustion of fuels, that is a strong oxidant. It irritates airways and worsen respiratory diseases and is an important ozone precursor. The maximum in average recommended is 25 ug/m3 in 8 hours.
SO2	is a gas with sharp odor and without color. The exposure to this gas is associated with asthma. The maximum in average recommended is 40 ug/m3 in a day.
VOCs	are compounds (usually human-made, such as industrial solvents), common ground-water contaminants. Since there are many different types, usually they are measured as TVOCs.
